# Supplementary material for: Has the Prevalence of Childhood Obesity in Spain Plateaued? A Systematic Review and Meta-Analysis
Source: Int J Environ Res Public Health. 2022 Apr 26;19(9):5240. doi: 10.3390/ijerph19095240 (PMC9105543; doi:10.3390/ijerph19095240)
Supplement: Supplementary file 1 [file ijerph-19-05240-s001.zip › ijerph-1653318-supplementary.pdf]

Figure S1. Flowchart for the studies included in the systematic review following PRISMA 2020.

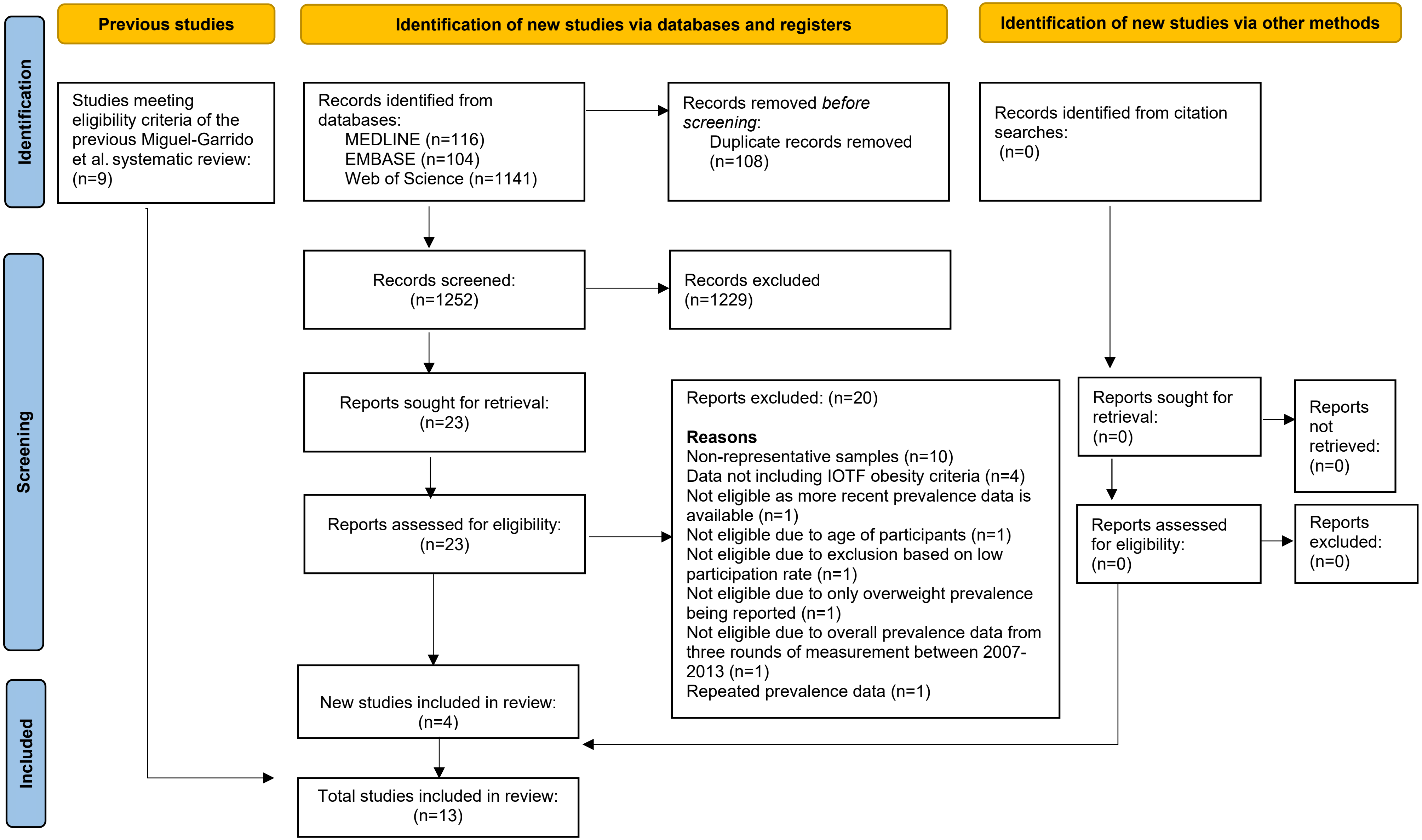

Supplementary Tables

Table S1. List of excluded studies in the systematic review and meta-analysis, with reasons.

Study	Reasons
**Previous studies from Garrido-Miguel M et al. 2019**	
Martin JJ et al. 2008	The sample was a non-representative sample of children from Oviedo city (convenience sample).
Aguilar Cordero MJ et al. 2011	Aguilar Cordero et al. did not provide information about the total (both sexes) prevalence of overweight or obesity in children (9–12 years). In addition, they did not report the number of boys or girls aged between 9 and 12 years.
Alvarez Caro F et al. 2011	The sample was a non-representative sample of children from Avilés city (convenience sample).
González Garcia A et al. 2015	The sample was a non-representative sample of children from Ciudad Real and Cuenca regions because 90% of the schools recruited (19/21) were public schools.
Gomez Santos SF et al. 2015	The sample was a non-representative sample of children (convenience sample of 14 municipalities). Thao Child Health Programme's main objective was healthy lifestyle promotion to children and their families.
ALADINO Study 2015	Excluded. We have included the latest prevalence data from ALADINO Study 2019.
**New studies excluded after full-text reading in the updated systematic review until December 2021**	
Spinelli A et al. 2019	Prevalence data using 3 rounds of COSI study (2007–2008; 2009–2010; 2012–2013)
Garcia-Solano M et al. 2021	Excluded. We included the latest prevalence data from ALADINO Study 2019
Buoncristiano M et al. 2021	IOTF definition of obesity was not used.
Ortiz-Marron H et al. 2018	The sample was a non-representative sample of children from Community of Madrid (used a Primary Care Physicians Sentinel Network to recruit children).
Yanez Ortega JL et al. 2019	The sample was a non-representative sample of children from Castilla y León region (used a Primary Care Physicians Sentinel Network to recruit children).

Garrido Miguel M et al. 2020	The sample was a non-representative sample of children from Cuenca region (randomized trials).
Ajejas-Bazan MJ et al. 2018	Authors did not include prevalence data stratified per age-groups (Age range 2–15 years).
Lopez-Gil JF et al. 2020	IOTF definition of obesity was not used.
Albadalejo R et al. 2019	Only overweight category was reported.
Jiménez Candel MI et al. 2021	The sample was a non-representative sample of children from Murcia city (convenience sample from one geographical area).
Falero Gallego MP et al. 2020	The sample was a non-representative sample of children from Castilla La Mancha region (convenience sample from one health area: La Mancha Centro)
Sanchez Campayo E et al. 2021	The sample was a non-representative sample of children (community-based intervention study to change food behavior in a town from Community of Madrid).
Lane JC et al. 2020	IOTF definition of obesity was not used.
Perez-Rios M et al. 2018	Repeated prevalence data (see another study selected in the current systematic review by the same authors).
Vaquero Alvarez M et al. 2019	The sample was a non-representative sample of children (small suburb from Córdoba region).
Heras Gonzalez L et al. 2020	The sample was a non-representative sample of children from Granada and Malaga regions (no mention of random sampling)
Calderon Garcia A et al. 2019	The sample was a non-representative sample of children from Community of Madrid (no mention of random sampling)
Fernandez Iglesias R et al. 2021	The sample was a non-representative sample of children from Asturias (Quota sampling, because they do not comprehensively describe stratified sampling).
Serral Cano G et al. 2019	IOTF definition of obesity was not used.
Manios Y et al. 2018	Authors considered non-eligible schools all those with a low participation rates (introducing a selection bias).

References

Garrido-Miguel M, Cavero-Redondo I, Álvarez-Bueno C, et al. Prevalence and Trends of Overweight and Obesity in European Children From 1999 to 2016: A Systematic Review and Meta-analysis. JAMA Pediatr 2019;173:e192430.

Martín JJ, Hernández LS, Gonzalez MG, Mendez CP, Rey Galán C, Guerrero SM. Trends in childhood and adolescent obesity prevalence in Oviedo (Asturias, Spain) 1992-2006. Acta Paediatr 2008;97:955-8.

Aguilar Cordero MJ, González Jiménez E, García García CJ, et al. [Obesity in a school children population from Granada: assessment of the efficacy of an educational intervention]. Nutr Hosp 2011;26:636-41.

Alvarez Caro F, Díaz Martín JJ, Riaño Galán I, Pérez Solís D, Venta Obaya R, Málaga Guerrero S. Factores de riesgo cardiovascular clásicos y emergentes en escolares asturianos [Classic and emergent cardiovascular risk factors in schoolchildren in Asturias]. An Pediatr (Barc) 2011;74:388-95.

González García A, Álvarez Bueno C, Lucas de la Cruz L, et al. [Prevalence of thinness, overweight and obesity among 4-to-6-year-old spanish schoolchildren in 2013; situation in the european context]. Nutr Hosp 2015;3:1476-82.

Gómez Santos SF, Estévez Santiago R, Palacios Gil-Antuñano N, et al. Thao-child health programme: community based intervention for healthy lifestyles promotion to children and families: results of a cohort study. Nutr Hosp 2015;32:2584-7.

García-Solano M, Gutiérrez-González E, López-Sobaler AM, Ruiz-Álvarez M, Bermejo López LM, Aparicio A, García-López MA, Yusta-Boyo MJ, Robledo de Dios T, Villar Villalba C, Dal Re Saavedra MÁ. Situación ponderal de la población escolar de 6 a 9 años en España: resultados del estudio ALADINO 2019 [Weight status in the 6- to 9-year-old school population in Spain: results of the ALADINO 2019 Study]. Nutr Hosp. 2021 Oct 13;38(5):943-953.

Spinelli A, Buoncristiano M, Kovacs VA, et al. Prevalence of Severe Obesity among Primary School Children in 21 European Countries. Obes Facts 2019;12:244-58.

García-Solano M, Gutiérrez-González E, López-Sobaler AM, et al. Weight status in the 6 to 9 year-old school population in Spain: Results of the ALADINO 2015 study. An Pediatr (Engl Ed) 2021;94:366-76.

Buoncristiano M, Williams J, Simmonds P, et al. Socioeconomic inequalities in overweight and obesity among 6- to 9-year-old children in 24 countries from the World Health Organization European region. Obes Rev 2021;22 Suppl 6:e13213.

Ortiz-Marrón H, Ortiz-Pinto MA, Cuadrado-Gamarra JI, et al. Persistence and Variation in Overweight and Obesity Among the Pre-school Population of the Community of Madrid After 2 Years of Follow-up. The ELOIN Cohort. Rev Esp Cardiol (Engl Ed) 2018;71:902-9.

Yáñez-Ortega JL, Arrieta-Cerdán E, Lozano-Alonso JE, et al. Grupo de Trabajo para la Investigación de Sobrepeso, Obesidad y Desarrollo Infantil en Castilla y León; Grupo de Trabajo para la Investigación de Sobrepeso, Obesidad y Desarrollo Infantil en Castilla y León. Prevalence of overweight and obesity in child population. A study of a cohort in Castile and Leon, Spain. Endocrinol Diabetes Nutr (Engl Ed) 2019;66:173-80.

Garrido-Miguel M, Martínez-Vizcaíno V, Herráiz-Adillo Á, et al. Obesity and thinness prevalence trends in Spanish schoolchildren: are they two convergent epidemics? Eur J Public Health 2020;30:1019-25.

Ajejas Bazán MJ, Jiménez Trujillo MI, Wärnberg J, Domínguez Fernández S, López de Andrés A, Pérez Farinós N. Differences in the prevalence of diagnosis of overweight-obesity in Spanish children according to the diagnostic criteria set used. Gac Sanit 2018;32:477-80.

López-Gil JF, López-Benavente A, Tárraga López PJ, Yuste Lucas JL. Sociodemographic Correlates of Obesity among Spanish Schoolchildren: A Cross-Sectional Study. Children (Basel) 2020;7(11):201.

Albaladejo R, Villanueva R, Astasio P, Ortega P, Santos J, Regidor E. Sports facilities, socio-economic context and overweight among the childhood population in two southern European cities: a cross sectional study. BMC Pediatr 2019;19:307.

Jiménez Candel MI, Carpena Lucas PJ, Mondéjar Jiménez J, García Pérez R, Gómez Navarro AJ. [The influence of a healthy lifestyle on body mass index in a sample of 12-14 year old individuals in Murcia (Spain)]. An Sist Sanit Navar 2021;44:33-40.

Falero Gallego MP, González González A, Muñoz Serrano A, Moreno Manzanaro I, Arias Arias A, Redondo González O. Excess weight in La Mancha-Centro schoolchildren by different criteria Nutr clín diet hosp 2020;40:73-82.

Sánchez Campayo E, Puga Giménez de Azcárate AM, Angulo Díaz-Parreño S, Ávila Torres JM, Varela-Moreiras G, Partearroyo T. Waist circumference as a prognostic index of childhood abdominal obesity: findings in the Spanish population. Nutr Hosp 2021;38:85-93.

Lane JC, Butler KL, Poveda-Marina JL, et al. Preschool Obesity Is Associated With an Increased Risk of Childhood Fracture: A Longitudinal Cohort Study of 466,997 Children and Up to 11 Years of Follow-up in Catalonia, Spain. J Bone Miner Res 2020;35:1022-30.

Pérez-Ríos M, Santiago-Pérez MI, Malvar Pintos A, Suanzes Hernández J, Hervada Vidal X. [How many kids are there with excess weight? What information should be published?]. Rev Esp Salud Publica 2018;92:e201806026.

Vaquero-Álvarez M, Romero-Saldaña M, Valle-Alonso J, Llorente Cantarero FJ, Blancas-Sánchez IM, Fonseca Del Pozo FJ. [Study of obesity in a rural children population and its relationship with anthropometric variables]. Aten Primaria 2019;51:341-9.

Heras-Gonzalez L, Latorre JA, Martinez-Bebia M, Gimenez-Blasi N, Olea-Serrano F, Mariscal-Arcas M. Sugar consumption in schoolchildren from southern Spain and influence on the prevalence of obesity. PLoS One 2020;15(11):e0242602.

Calderón García A, Marrodán Serrano MD, Villarino Marín A, Martínez Álvarez JR. [Assessment of nutritional status, and habits and food preferences in a child-youth population (7 to 16 years) of the Community of Madrid]. Nutr Hosp 2019;36:394-404.

Fernández-Iglesias R, Álvarez-Pereira S, Tardón A, Fernández-García B, Iglesias-Gutiérrez E. Adherence to the Mediterranean Diet in a School Population in the Principality of Asturias (Spain): Relationship with Physical Activity and Body Weight. Nutrients 2021;13:1507.

Serral Cano G, Bru Ciges R, Sánchez-Martínez F, Ariza Cardenal C. [Overweight and childhood obesity according to socioeconomic variables in third grade school-age children in the city of Barcelona]. Nutr Hosp 2019;36:1043-8.

Manios Y, Androutsos O, Katsarou C, et al. Prevalence and sociodemographic correlates of overweight and obesity in a large Pan-European cohort of preschool children and their families: the ToyBox study. Nutrition 2018;55-56:192-8.

Table S2. Search strategy for Medline.

	**Search terms**
**1. Population**	(Children OR Childhood OR Schooler OR Preadolescent OR “School aged” OR “School-aged”)
**2. Outcome**	(Obesity OR Overweight OR “Body composition” OR “Body constitution” OR “Weight status” OR anthropometr*)
**3. Study design**	(Prevalence OR Trend OR Epidemiolog*)
**4. Types of studies**	(Observat* OR “cross-sectional” OR longitudinal NOT (survey* OR review))
**5. Location**	Spain
	1 AND 2 AND 3 AND 4 AND 5

Truncation symbol: ∗ = all possible word endings included.
